# Computational Methods for MicroRNA Target Prediction

**DOI:** 10.3390/genes5030671

**Published:** 2014-08-22

**Authors:** Semih Ekimler, Kaniye Sahin

**Affiliations:** Molecular Biology and Genetics Department, Faculty of Science, Istanbul University, Vezneciler Fatih, 34134 Istanbul, Turkey; E-Mail: kaniye@istanbul.edu.tr

**Keywords:** miRNA, bioinformatics, target prediction, gene expression regulation, computational methods

## Abstract

MicroRNAs (miRNAs) have been identified as one of the most important molecules that regulate gene expression in various organisms. miRNAs are short, 21–23 nucleotide-long, single stranded RNA molecules that bind to 3' untranslated regions (3' UTRs) of their target mRNAs. In general, they silence the expression of their target genes via degradation of the mRNA or by translational repression. The expression of miRNAs, on the other hand, also varies in different tissues based on their functions. It is significantly important to predict the targets of miRNAs by computational approaches to understand their effects on the regulation of gene expression. Various computational methods have been generated for miRNA target prediction but the resulting lists of candidate target genes from different algorithms often do not overlap. It is crucial to adjust the bioinformatics tools for more accurate predictions as it is equally important to validate the predicted target genes experimentally.

## 1. Introduction

Since the discovery of microRNAs in the early 1990s, the view of the regulation of gene expression has started to change. The first identifications of the endogenous short regulatory RNAs in *Caenorhabditis elegans* [1] and the 7–8 nucleotide-long motifs in the 3' untranslated regions (3' UTRs) of *Drosophila melanogaster* miRNAs [2] revealed that expression of genes are under control of small noncoding RNAs (sncRNAs) which bind to mRNAs and repress their expression post-transcriptionally. Later on, these small regulatory RNAs were affiliated as microRNAs (miRNAs) and the regions in the mRNAs, as their target sites [3,4]. The regulation of gene expression mediated by miRNAs involves the processing of hairpin transcripts into ~22 nucleotide-long RNAs, association with Argonaute proteins which guide them to their target sites on mRNAs, and ultimately, repression of gene expression via mRNA degradation and/or translational inhibition. In the animal kingdom different genomes maintain diverse numbers of miRNA genes [5] and miRNAs affect the expression of genes in almost every developmental and physiological processes [6]. Furthermore, miRNAs are being used by industrial companies both as targets and therapeutic agents in order to generate new treatment methods for diseases [7].

The main obstacle in miRNA research is to detect the specific sequences on target genes that miRNAs are fully or partially complementary [8] and to define how miRNAs recognize those sequences considering that the size of miRNAs exhibits insufficient information for specificity. Additionally, partial complementarity usually comprises a wide range of possible target genes which means that a single miRNA could regulate multiple genes. Among those some of them are not necessarily the actual targets of the miRNA [9]. In order to overcome this complexity and to predict the target genes precisely, several algorithms have been generated and are still being developed. The prediction methods that use these algorithms are diverse and the algorithms can be improved depending on the approach of the researchers and the performance demand [4]. In this article we will discuss the difficulties in predicting miRNA target genes based on the nature of miRNA function and the regulation of miRNA expression. We will review the current target gene prediction methods with their advantages and drawbacks, and finally we will mention up-to-date developments for more precise target prediction.

## 2. MicroRNAs: Biogenesis and Function

MiRNAs are short single-stranded RNA molecules which are transcribed from endogenous genes by RNase polymerase II initially as long precursors (pri-miRNAs). Pri-miRNAs vary from 70 to 100 nucleotides in size and fold into a hairpin-loop structure consisting paired bases with several mismatches and bulges [10]. The hairpin-loop structures are first excised in the nucleus by an RNase III enzyme Drosha and its cofactor DGCR8/Pasha to generate pre-miRNAs [11]. Pre-miRNAs are then transported from nucleus to cytoplasm by Ran-GTP-dependent Exportin-5 [12] protein through the nuclear pore complexes embedded in the nuclear membrane [13]. The maturation of pre-miRNAs is mediated by another RNase III, Dicer, in the cytoplasm. Dicer cuts the pre-miRNAs and generates 21–23 nucleotide-long double-stranded RNAs which are mature miRNAs and ready to function [14]. Following the unwinding of double helix structure, mature miRNAs are joined to a ribonucleoprotein complex which is called “RNA-induced silencing complex” (RISC) [15]. In this complex, one strand is retained as a functioning miRNA and the other strand is eliminated. The functioning strand has been shown to have a thermodynamically more stable 5' end which remains in the complex [16]. It has been shown that in the animal kingdom the pairing between miRNA and 3' UTR of target mRNA is partial and most common pattern is the perfect complementarity of the target mRNA to the 5' end of miRNA between the second and seventh nucleotides which is called the “seed” region. The pairing between the seed region and the target mRNA is essential and adequate for the efficient miRNA-mediated regulation of gene expression [17].

The biological processes of miRNA-regulated gene expression are various including cell proliferation, cell differentiation, apoptosis, signal transduction, immune response, stress resistance, fat metabolism, insulin secretion and hematopoiesis [18]. In 1993, the first miRNAs, lin-4 and let-7 were identified as regulators for the transition of developmental stages in *C. elegans* [1]. Mainly miRNAs influence the expression of their target genes in a negative manner via mRNA degradation and translational repression but there is also enough data supporting that they enhance the expression of genes [19]. The mRNA degradation pathway of miRNAs is well understood based on their similar behavior to siRNAs that cleave their target mRNAs from tenth or eleventh nucleotides of the duplex [20]. The level of complementarity between the miRNA and its target mRNA has a role in selecting the silencing pathway. It has been understood that near-perfect base pairing leads to mRNA degradation and base pairing with mismatches and bulges causes translational inhibition [21]. According to thermodynamic experiments on miRNA and target binding, a correlation has been found between the translational repression and the required free energy for binding of the seed region [17]. In plants, however, miRNAs usually induce the degradation of their target transcripts via perfect or near-perfect complementarity base pairing [22]. Although it is not very clear how miRNA-mediated translational repression occurs, blocking the transport of ribonuclear particles to ribosomes, arresting and delaying protein translation on ribosomes or interfering the formation of protein complexes near ribosomes can be achieved by miRNAs in order to alter protein levels posttranscriptionally [23].

### Features of miRNAs that Affect the Target Recognition

Inside the cell there are several features that regulate the target recognition of miRNAs. The first and the most recognized feature is the sequence specificity of miRNAs to 3' UTR of mRNAs. Even though this feature is quite limited, the AU residues in target sites increase the accessibility of the miRNAs to form duplexes [8]. The target sites are also prone to exclude the sequences adjacent to the stop codon at the end of the mRNA which are covered by ribosomes [24]. The newest studies on target identification have suggested that coding regions of mRNAs can also include target sites for miRNAs. These studies are based on reporter assays and are supported by biochemical methods for genome-wide identification of binding sites and RISC components [25,26,27]. Most of these studies, however, have been conducted excluding the analysis of 3' UTR targets but Fang and Rajewsky have succeed to analyze the combined effects of seed regions in both coding region and 3' UTRs by using large-scale and genome-wide miRNA misexpression data. They stated that target sites solely in the coding region are usually less efficient but they help to increase the effect of miRNAs when there is also a 3' UTR target region on the mRNA [28]. As a rule, multiple target sites increase the effectiveness of regulation for a particular miRNA but when the target sites have overlapping sequences the regulation can be compromised. It has been demonstrated that multiple target sites can have suppressing and rarely enhancing activities [24,29,30].

## 3. Methods for Identifying miRNA Targets

From the moment of understanding the effects of miRNA regulation of genes, studies have been focused on predicting the accurate target genes for miRNAs and considerable number of methods has been identified so far. These methods diversify from small-scale genetic methods and high-throughput biochemical processes for target sequence isolation to computational prediction methods [31]. The most demanding complication in predicting functional miRNA targets is the lack of available experimental evidence to validate computational methods [9].

Genetic methods use phenotypic suppression tests for identifying miRNA target sequences. Generally, an miRNA mutant type which has a loss-of-function phenotype is constructed by traditional mutagenesis or RNAi mechanism and the candidate mRNAs are screened for suppression. In the mutant strains, target genes are repeatedly upregulated. The recognized advantage of the genetic methods is the direct relation between the identified target gene and a specific physiological process in the cell which is regulated by the miRNA. However, these methods are not qualified for distinguishing indirect targets of miRNAs or determining multiple targets that cause the same phenotype [9].

Biochemical approaches for identifying miRNA targets are more sophisticated than genetic methods and they are usually associated with bioinformatics analyses. They have augmented the sensitivity in identifying target mRNAs endogenously. First attempts were based on immunopurification of miRNA ribonucleoprotein (miRNP) complexes, isolation of related mRNAs, and microarray analysis of target transcripts subsequently [26,32]. Recently, ultraviolet crosslinking and immunoprecipitation coupled with deep sequencing (CLIP-seq) or high-throughput sequencing coupled with ultraviolet crosslinking and immunoprecipitation (HITS-CLIP) are used for isolating target endogenous mRNAs and next-generation sequencing tools provide high-resolution sequence data for the target mRNAs [27,33]. The biochemical approaches provide genome-wide targets of miRNAs and the exact binding sequences located in the mRNAs which augment the knowledge about seed pairing, conservation and structure. They also bring new insight into prediction of binding sites such as coding regions of mRNAs. The drawback of CLIP-based methods is that the identified binding sites are not functionally definite and further investigations such as genome-wide transcriptome profiling with microarrays or RNA sequencing (RNA-seq) and quantitative proteomic platforms with stable isotope labelling with amino acids in culture (SILAC) are needed [34].

Computational methods have been based on algorithms that are generated by the physical properties of miRNA regulation, subsequently as perfect Watson-Crick complementarity between the first 2–7 nucleotides of miRNA and the 3' UTR of mRNAs, evolutionary conservation, and secondary structures of 3' UTRs based on AU content. These methods generate many false positive predictions and the results need to be experimentally validated. In order to test computational predictions for functional regulation, heterologous reporter genes are fused to target 3' UTRs; however, the function of predicted sites can still remain questionably ambiguous [9].

## 4. Bioinformatics Tools for Predicting miRNA Targets

Using bioinformatics tools for predicting target genes is relatively a new approach regarding the challenging prediction algorithms and complexity of miRNA-target interactions. Prior to performing high-throughput experiments it is critically important to determine miRNA targets by computational methods and the complementarity between miRNA and target mRNA has been the fundamental advantage for computational analysis [35]. In order to generate computational algorithms for identifying miRNAs and their targets, first Watson-Crick complementarity in the seed region between the miRNA and the target is analyzed, then sequence comparison between species is investigated for evolutionary conservation. Finally, thermodynamic favorability of the miRNA-mRNA duplex is analyzed by free energy calculations and the site accessibility is monitored to determine the secondary structure of the duplex [36].

Computational methods for miRNA target prediction usually start with simple algorithms that depend on Watson-Crick base pairing between the seed region of the miRNA and the 3' UTR regions of mRNA. The Ensembl database comprises 3' UTR regions for human, mouse, and zebrafish genomes that are extracted from alignments of cDNAs and expressed sequence tags [37]. However, algorithms based on sequence complementarity have low accuracy and high number of false positive results [8]. In order to reduce the false positive results, conservation analysis has been added to algorithms, and the binding sites that are not conserved among species are filtered out. The use of conservation of predicted binding sites among orthologous 3' UTRs is not completely precise when the closely related organisms such as humans and chimpanzees are used. Conservation analysis with comparatively less related species gives more relevant results [4]. Experimental and computational methods have also demonstrated that W:U wobble base pairing is minimum in the seed region and it is thought that this feature reduces the silencing efficiency. Even though the binding in the seed region is highly strong, less strong bindings and mismatches exist in the central region and the 5' end of the miRNA [38]. The weak binding in those regions is significantly important in regulating the expression of target mRNA. The interactions between other miRNAs and the secondary structure of target mRNA also establish great variety in effects of miRNAs on target mRNAs [39]. Different methods have been developed for computational target prediction (Table 1). 

**Table 1:** The main algorithms for computational target prediction.

Algorithm	Website	Last Update	Input	Organisms	Features	References
miRanda	www.microrna.org	August 2010	miRNA name, miRNA sequence, gene name	human, rat, mouse, fly, worm	seed match, conservation, free energy	Enright *et al.*, 2003 [40] John *et al.*, 2004 [23]
TargetScan	www.targetscan.org	June 2012	miRNA name, miRNA family, gene name	mammals, fly, worm	seed match, conservation	Lewis *et al.*, 2005 [41] Grimson *et al.*, 2007 [24] Friedman *et al.*, 2009 [42] Garcia *et al.*, 2011 [43]
DIANA-microT-CDS	www.microrna.gr/microT-CDS	July 2012	miRNA name, gene name, Ensembl ID	human, mouse, fly, worm	seed match, conservation, free energy, site accessibility, target-site abundance	Maragkakis *et al.*, 2009 [44] Reczko *et al.*, 2012 [45] Paraskevopoulou *et al.*, 2013 [46]
PicTar	pictar.mdc-berlin.de	March 2007	microRNA name, gene name	vertebrate, fly, worm, mouse	seed match, pairing stability	Lall *et al.*, 2006 [47]

The Stark method which Stark and his colleagues generated was the first miRNA target prediction tool for *Drosophila melanogaster* [48]. The search tool was designed for identifying the reverse complement of miRNA sequences and for enabling G:U wobble matches. The 3' UTRs resulted from prediction algorithm were filtered based on conservation analysis between *Drosophila pseudoobscura* and *Anopheles gambiae*. Subsequently, the target sites were scored and used as an input for the algorithm [49].

The miRanda algorithm was generated after the Stark method and it hunts highly overlapped base pairing in the 3' UTRs for identifying potential binding sites [40]. The algorithm gives higher scores for complementarity in the 5' end of the miRNA than the 3' end which leads to better results for the seed regions that have perfect or almost perfect match. The results are evaluated for thermodynamic stability and conservation [50]. Even though the prediction methods of Stark and miRanda are similar the scoring approaches are different and only 40% of predicted miRNA targets are common for both algorithms which shows that small modifications in the algorithm leads to significant alterations in the results [17].

The TargetScan algorithm, on the other hand, uses a different approach from the other two methods. It searches for perfect complementarity in the seed region and beyond [4]. Complementarity outside the seed region filters out the false positives more efficiently prior to prediction. The data from conservation analysis derived from orthologous 3' UTRs are also used as an input early in the process. Afterwards thermodynamic stability is tested to filter predicted target sites [50]. TargetScan also was the first miRNA target prediction tool for human genome [4].

The PicTar algorithm firstly uses conservation data from 3' UTRs of multiple species as input data and looks for the alignment of complementary seed regions. The resulting binding sites are then checked for thermodynamic stability and each result is scored based on the Hidden Markov Model maximum-likelihood fit approach. PicTar is the first algorithm for analyzing miRNAs and their targets in co-expression at specific time and place [51]. A novel database, doRiNA, provides miRNA target region predictions computationally for human, mouse, and worm, based on the most updated PicTar predictions [52].

The Diana-microT algorithm has a larger frame for scanning complementarity and focuses on coding regions of target mRNAs to increase sensitivity and accuracy. It also calculates and uses the free energy of binding sites as an input data for the prediction [53].

Current online databases for miRNAs provide data for miRNA expressions, miRNA sequences, experimentally validated or putative target mRNAs and their sequences, genomic locations, and expression levels in various tissues or cell lines [54]. Most of the databases derived from those algorithms let users adjust threshold levels to suit their experimental analysis more specifically and more precisely. All of the algorithms have been experimentally validated and they have been merged as DIANA-TarBase, miRTarBase, miRecords and StarBase in order to retrieve, create and analyze new data. A cross check between multiple algorithms is necessary for accurate true positive target predictions and for providing commonly agreed results from different algorithms and inter-method and inter-database comparisons [55,56].

### Recent Strategies for Computational Target Prediction

While the first strategies for target prediction focused mainly on the base-pairing, recent methods have increased the accuracy by using CLIP-seq based expression studies, single nucleotide polymorphisms (SNPs) at the target regions, or detecting miRNA family clusters. Many bioinformatics resources have been developed for miRNA target identification using CLIP-seq data such as Piranha [57] and CLIPZ [58]. Piranha uses a zero-truncated negative binomial regression model to cover external data for guiding the target identification. On the other hand, CLIPZ presents a database and the analysis of binding sites of the RNA-binding proteins which are experimentally identified.

The required data for the computational analysis of the target sites in coding sequences are being derived from mammalian tissues using high-throughput immunoprecipitation and sequencing methods. One of the most important progresses in this area is the work of Hafner and his colleagues. They have identified the miRNA-containing ribonucleoprotein complexes and the sequences of the associated RNAs via immunoprecipitation and PAR-CLIP methods. They have demonstrated that miRNAs have approximately equal tendency in binding to 3' UTRs and coding sequences [27]. Reczko and colleagues have developed an algorithm for miRNA target prediction both in 3' UTRs and coding sequences using data from Hafner *et al.*’s work. They have identified 12% of the downregulated genes as additional targets of miRNAs which have target sites in the coding sequences [45]. Another web server that uses CLIP research data is STarMir, which makes predictions of miRNA binding sites at target RNAs using an implemented logistic prediction model [59].

Expression-based methods have been used to reveal miRNA-mRNA interactions. Radfar and his colleagues developed a new computational method, InMiR, based on linear-Gaussian model for the targets of the intronic miRNAs and the expression profiles of the host genes. They classified the intronic miRNAs in three groups: the first group includes miRNAs that are strictly regulated by their host gene, the second group consists of miRNAs that use the same promoter with the host gene but are regulated by other miRNAs, and finally the third group has miRNAs that interact with completely independent promoters. This method has a success rate of almost two times more true positives than the methods that use solely the correlation [60].

Single nucleotide polymorphisms (SNPs) are another important aspect in regulation of gene expression. SNPs at regulatory sequences have an effect in transcriptional and posttranscriptional level. Regarding this phenomenon, SNPs at miRNA target sites can also alter miRNA function and a certain number of databases such as polymiRTS [61], Patrocles [62], and miRdSNP [63], have been built to help identifying the impacts of SNPs in miRNA and target interaction. polymiRTS presents the polymorphisms in putative miRNA target regions and identifies the related effects at the locus. Patrocles database collects polymorphisms at 3' UTRs in seven vertebrate organisms. miRdSNP, however, consists of dSNPs from human genome that have been registered in PubMed. With a very powerful web interface and useful search engine, this database helps to demonstrate distances between SNPs and putative miRNA target regions from the most frequently used algorithms TargetScan [41] and PicTar [51].

Instead of focusing on miRNA target identification, Zou *et al.* have paid attention to miRNA family identification. They have developed two sources for miRNA classification: miRBase and Rfam. Their aim is to accumulate all the related biological data and to improve medical implications of miRNAs [64].

Prediction of miRNA target genes is a challenging process and these processes usually result with excessive number of genes. The algorithms for miRNA target prediction provide distinct targets, and resulting overlapping targets are less than expected [65]. Generally, the large-scale predictions of targets in the genome aim the comparison of specifity and sensitivity [66]. The sensitivity and specifity of current algorithms vary and among them TargetScan, PicTar, and miRanda are identified as best methods after the evaluations [67]. Additionally very few algorithms are capable of integrating data from different studies to investigate miRNA function. Hence, it is vital to generate accurate computational methods to determine the functional miRNA-target interactions using miRNA target predictions and gene expression profiling. Integration of miRNA target prediction and gene expression studies are carried out based on the acknowledged criteria that upregulation of a specific miRNA decreases the expression of its target and downregulation of a specific miRNA increases the expression of its target [66]. Currently, there are several computational tools for combining the target prediction and expression analysis, such as miRBase, Argonaute, miRNAmap, and miRGen [68]. ComTAR is a very useful tool for predicting and characterizing the conserved microRNA targets in plants [69].

For the integration of data from computational target predictions and experimental expression analysis numerous software programs are developed including miRGator [70], SigTerms [71], and TopKCEMC [72].

## 5. Conclusions

The miRNA target prediction is dependent on computational methods and recent developments have increased the pace of generating new strategies making the future of miRNA target prediction promising. The algorithms have been progressively modified for more accurate results based on the expanding understanding of molecular mechanism of miRNA regulation. Also, high-throughput new generation sequencing technologies has been altering the current vision of posttranscriptional regulation of gene expression by sequencing 5' ends of miRNAs directly. Finally, online databases are improved and extended for more specific search options in order to provide researchers better quality information by retrieving more research-oriented data.

In this review, the common features of the miRNA target prediction methods have been summarized. The newly-developed strategies that have integrated different target prediction tools to provide more sophisticated data including miRNA expression profiles, gene function, and gene ontology have been described. Even though current databases and bioinformatics tools help researchers to investigate new miRNA targets, it is considerably important to use the most suitable prediction tool to obtain more accurate data based on the version, maintenance, and data utilization of each tool. With the current pace of data processing strategies and the increasing data from genome-wide computational and experimental approaches, miRNA-target predictions will become more useful for scientific research and clinical implementations.
